# Human dignity and ontological foundations: a philosophical perspective for the health professions

**DOI:** 10.1186/s13010-025-00207-w

**Published:** 2026-01-08

**Authors:** Patrícia Frantz, Francisca Rego, Stela Barbas

**Affiliations:** 1 University of Porto, Porto, Portugal; 2 Universidade do Sul de Santa Catarina, Palhoça, Brazil

**Keywords:** Human dignity, Bioethics, Metaphysics, Medical ethics, Medical philosophy

## Abstract

The question of what it means to be human remains one of the most fundamental inquiries in philosophy, with profound ethical implications, particularly in healthcare. This paper offers a conceptual framework for healthcare professionals by exploring the ontological status of the human being and the concept of personhood, grounded in classical metaphysical principles. Through a phenomenological, epistemological, axiological, and ontological lens, it proposes a unified understanding of human dignity that can inform and elevate clinical practice. While the dialogue between Greek philosophy and the Judeo-Christian tradition established a robust and enduring notion of dignity, and Kantian ethics reinforced the centrality of the human being as an end in itself, the increasing compartmentalization of knowledge—though fruitful in some respects—has obscured the integral vision of the human person. In the medical field, this fragmentation can diminish awareness of the relational, existential, and spiritual dimensions essential to humane care. In response, this paper reaffirms the relevance of philosophical anthropology for medical ethics. It contends that safeguarding human dignity amid contemporary scientific and technological challenges requires returning to an ontological vision of the person-one that transcends functionalist and reductionist models and restores the human being to the center of healthcare. By doing so, it offers professionals a deeper foundation for ethical discernment and compassionate practice.

## Introduction

What does it mean to be human? Far from being a merely theoretical question, this inquiry lies at the heart of some of the most urgent debates in contemporary bioethics. In contexts such as end-of-life decisions, the treatment of persons with disabilities, and the ethical challenges raised by emerging technologies, the answer to this question directly shapes both clinical practice and public policy. While the notion of “human dignity” has become a central pillar of bioethical discourse, it is often employed without a clear ontological foundation—leading to tensions, contradictions, and, at times, ethical ambiguities.

This article argues that the dignity of the human being cannot be reduced to functionalist criteria, such as self-consciousness or rational agency, as proposed by thinkers like Peter Singer [1] and Michael Tooley [2]. Instead, we adopt an ontological approach in which dignity is inherent to the human being as such—prior to and independent of any capacities or social recognition. This is not a mere conceptual preference, but a fundamental ethical orientation. The way we define what it means to be human has direct implications for how we care for the most vulnerable among us.

In contemporary bioethics, the concept of the “person” is often separated from that of the “human being”—a distinction that has justified the exclusion of certain individuals from full moral status. Thinkers such as H. Tristram Engelhardt argue that only beings capable of rational moral agency qualify as persons, thereby grounding moral worth in functional criteria [3]^1^. Developed within analytical philosophy, this distinction has led to influential arguments denying moral status to infants, individuals with advanced dementia, or patients in persistent vegetative states. Within this framework, the human being—reduced to a biological substrate—becomes ethically relevant only if specific cognitive thresholds are met.

This article rejects such fragmentation. Following a Thomistic perspective, we argue that the human being is a *substantia individua rationalis*—an individual substance of a rational nature, whose dignity is grounded not in what they can do, but in what they are. This definition, originally articulated by Boethius [4] and later refined by Aquinas [5], affirms the unity of body and soul, matter and form, nature and transcendence. It stands in stark contrast to both Cartesian dualism, which severs mind from body, and to contemporary physicalism, which denies any spiritual or immaterial dimension to the human person.

This ontological understanding of the person has deep roots in both classical philosophy and the Judeo-Christian tradition. For the Greeks, human beings were defined by their rationality and capacity for contemplation [6]. In the biblical view, they are made in the image and likeness of God^2^—entrusted with stewardship over creation and called to communion. These two currents converged to form a vision of the human being as a relational, moral, and transcendent being. Under this integrative model, anthropology, ethics, and metaphysics are inseparable.

The modern era, however, introduced a series of ruptures. With the rise of Cartesian thought, matter became the exclusive domain of science, while the soul was increasingly relegated to theology or private belief. The Enlightenment, despite affirming human dignity, redefined it through the lens of rational autonomy [7]. The ethical catastrophes of the 20th century—including genocides and totalitarian regimes—have raised questions about the adequacy of purely procedural or secular frameworks when they operate independently of a substantive philosophical anthropology. The adoption of the Universal Declaration of Human Rights in 1948 was a necessary moral response, but its philosophical foundations remain limited. While the preamble refers to the “inherent dignity” and the “equal and inalienable rights” of all members of the human family, it does not offer a clear ontological grounding for these affirmations [8].

Meanwhile, scientific and technological developments—such as the mapping of the human genome—have vastly expanded our empirical knowledge of the human body [9]. Yet, as Max Scheler observed, the more science advances, the more elusive the meaning of “man” becomes (2008). Knowledge has multiplied, but understanding has fragmented. While the 20th century brought significant philosophical developments—particularly through existentialism and personalism—it also introduced a profound diversification of approaches to the human being [10]. The ethical and political upheavals of the post-war period, the ecological crisis, and the cultural transformations of the 1960s shifted attention toward new urgencies, often decentering the human from metaphysical reflection.

The rise of bioethics itself reflects this turn, as it sought to respond to novel situations without necessarily resolving the deeper anthropological questions. As the concept of “rights” expanded, so did the contradictions surrounding it—especially in matters such as abortion, euthanasia, and freedom of conscience—revealing an underlying fragility in our concept of the human. In this context, the call for an interdisciplinary philosophical anthropology is not nostalgic but urgent. As Blanca Castilla de Cortázar argues, we must reconstruct the concept of the human being with intellectual rigor and ethical clarity, drawing upon philosophy, science, and culture [11].

This article responds to that call by grounding the concept of dignity in a classical philosophical anthropology—particularly the Thomistic tradition—seeking to offer a deeper and more coherent framework for ethical reflection in healthcare. The aim is not to introduce a novel definition of the human person, but to recover a metaphysical understanding capable of sustaining the normative weight that contemporary bioethics often attributes to the notion of dignity. This interdisciplinary investigation engages philosophical, anthropological, and medical perspectives, highlighting a lacuna in the formation of healthcare professionals: namely, the absence of a robust philosophical foundation for understanding the human person.

Critics of the concept of dignity, such as Ruth Macklin [12], argue that it adds little to the principles already provided by autonomy, beneficence, and justice. Others, such as Singer, claim that dignity should apply only to “persons,” defined by functional criteria [1]. In contrast, this article reaffirms the centrality of dignity not as a derivative concept, but as a metaphysical property rooted in human nature. It contends that the erosion of this foundation weakens our ability to respond to the ethical challenges of our time.

The structure of the article follows a clear progression: grounded in the classical tradition, it begins with a reexamination of what it means to be human—through an inquiry into metaphysical principles, the faculties of the human being and the nature of the soul, the meaning of embodiment, and a brief account of human phenomenology and the historical development of the concept of person. It then contrasts ontological views of the person with functionalist and materialist perspectives, and finally turns to concrete implications in bioethics—arguing that a renewed metaphysical anthropology offers a more consistent and humane foundation for ethical action. The goal is not to provide a comprehensive history, but to recover the metaphysical depth of the human question, and to clarify what is ultimately at stake in contemporary debates on human dignity.^3^

## Metaphysical foundations according to the classical tradition

### Essence and substance

Rooted in the perennial insights of classical philosophy, our inquiry begins with a foundational distinction: that between essence and substance^4^. These are not mere terminological artifacts, but conceptual instruments that allow us to perceive the inner intelligibility of being. As such, they serve as the threshold through which the human being—indeed, any being—becomes discernible not only as a datum of experience, but as a bearer of meaning.

Aristotle defines the nature of a thing as its essence—that which makes a being what it is [13]. Essence is expressed through the definition of an entity, describing its fundamental characteristics and purpose. For instance, the essence of a chair is to serve as an object for sitting. In the case of the human being, essence refers to what defines us as human, such as the capacity to reason, love, create, and transcend. Boethius complements this understanding by asserting that the essence of a being is that which gives form through the “specific difference,” or what distinguishes it from other beings (1999). Thus, in this work, the term “nature” refers to this essence that renders something intelligible and confers its identity.

In the human being, composed of matter and form, both essence and the act of being coexist, with the latter serving as the principle of subsistence^5^. Human essence is tied to form (or soul, as we will later discuss), while the body represents the matter that actualizes this essence. The union of matter and form is what defines the human being as an individual substance—that is, a being that exists independently and sustains itself.

For Aristotle, substance is the stable and permanent core of a being, that which makes it “itself.” In the case of a human being, its substance is its nature, which remains constant despite changes over time. For example, even if a person undergoes physical or psychological changes, they remain the same substance throughout their life. In contrast, accidents are attributes that are not essential to the identity of the being but depend on the substance for their existence. For example, a person’s height or skin color are accidents: they may change without altering who the person is in essence.

The biblical metaphor found in Jeremiah—“Before I formed you in the womb, I knew you”^6^—can be philosophically interpreted as a reference to the idea that each human being possesses a unique essence or “formula” even before physical existence. This “formula” represents the immaterial potential of what the person can become, a concept Aristotle calls “potency.” This suggests that our existence, before being actualized, was a potentiality—whether in natural processes or within the divine plan of creation. The transition from potentiality to actuality occurs when this immaterial essence unites with matter, creating a unique human being endowed with countless potentialities.

### The theory of the four causes

For Aristotle, understanding something fully requires answering the question, *“Why does this exist?”* His theory of the four causes explains the existence and nature of all things [16]. Applied to the human being, it provides an integrated perspective of the self.

The material cause of the human being is the physical body, composed of genetic makeup and biological conditions—its tangible foundation. However, as Aristotle emphasizes, matter alone does not define what we are. The formal cause pertains to the essence of the self, responsible for identity and continuity over time. In humans, this is the soul, which organizes and animates the body, conferring unity and purpose. The soul, as the form of the body, is not a separate substance but the vital principle that gives meaning to matter, ensuring the persistence of identity despite physical and psychological changes. The efficient cause involves the external forces that shape a person—social interactions, cultural influences, education, and experiences—factors particularly relevant in healthcare, where they deeply impact physical and mental well-being. Lastly, the final cause refers to the ultimate purpose of human existence. For Aristotle, this is *eudaimonia*—not mere pleasure, but holistic fulfillment through virtue, self-knowledge, and the realization of potential [17]. In clinical practice, this notion aligns with promoting well-being and helping individuals find meaning and purpose, even in adversity.

The theory of the four causes provides an integrated model for understanding the human being in its multiple dimensions. For healthcare professionals, this means recognizing that the physical body (*material cause*) cannot be treated in isolation. It is essential to consider the *formal cause* (the patient’s identity and essence), the *efficient cause* (environmental and social factors shaping their life), and the *final cause* (their values and purpose). Such a holistic approach is critical for care that respects human dignity and promotes integral well-being, acknowledging the human being not merely as a biological organism but as a being endowed with transcendence and meaning.

### Mode of being

All beings possess distinct modes of being, classified by their level of ontological perfection. At the most basic level are inanimate beings, which lack life, followed by vegetative beings, capable of growth, nutrition, and reproduction^7^. Next are sensitive beings, which, like animals and humans, experience emotions and sensations. At the highest level are rational beings, uniquely endowed with *logos* (reason and language), whose intellect allows them to transcend material reality.

The fundamental distinction between natural beings (composed of matter and form) is whether or not they possess life. Living beings, unlike inert entities moved solely by external forces, have an internal principle that animates them. Life, therefore, is an intrinsic activity arising from within the being itself [15]. The perfection of a living being is measured by two key aspects: immanence and transcendence. Immanence refers to actions originating within and retained within the being—such as digestion, memory, or rational thought, the latter being the most profound and enduring [5]. Transcendence, in contrast, involves actions that extend beyond the being, allowing it to surpass its natural limits. This is most evident in human rationality, which enables the creation of ideas, art, and technology [15].

Additionally, living beings possess an internal principle of movement—their nature—which differentiates them from automatons that rely on external causes. Humans, in particular, exhibit a unique adaptability, inhabiting diverse environments due to their rationality. The perfection of a being is thus proportional to its capacity for immanence and transcendence: plants perform only vegetative actions, animals add sensitive functions, while humans integrate the intellective dimension, which is transcendence par excellence.

Finally, living beings are defined by unity and organicity. Their unity is evident in the cohesion of their parts, which function interdependently for the good of the whole. This does not imply uniformity—each part has a specific role, forming a dynamic system where diversity and cohesion harmonize to sustain life.

### Soul

Aristotle defines the human being as a rational animal, possessing a soul (*psyche*) capable of intelligence and reason [18]. The soul is the principle of identity and continuity, integrating body and soul into an inseparable unity. Even as the body undergoes physical changes, an essential permanence sustains the self throughout life, actualizing its potentialities until death.

Derived from the Latin *anima*, the term “soul” distinguishes living beings from inanimate ones. In Aristotle’s hylomorphic theory, all beings consist of matter (*hylé*) and form (*morphé*), with the soul as the substantial form that organizes and animates the body. Life, therefore, transcends mere biochemical processes, as it is guided by a vital principle that actualizes matter into a unified and ordered whole. As Lombo notes, reducing life to its material components overlooks its distinctive nature, which cannot be explained solely by subatomic interactions. Aristotle thus defines the soul as the first actuality of a natural body having life potentially, emphasizing that it is not a byproduct of bodily functions but their organizing principle.

For Aristotle, the body and soul do not exist as separate entities but form an integrated unity. The soul is the formal cause that organizes matter and directs it toward its purpose, ensuring that the body is not mere undirected matter and that the soul, in turn, can only operate within a physical reality. Unlike dualistic perspectives, this view maintains that the soul is simple (indivisible and non-composite), incorporeal (acting upon matter without being confused with it), non-spatial (not occupying physical space), and unique (each being has a single substantial form that integrates its entire organism).

Aristotle classifies the soul into three hierarchical levels. The vegetative soul, present in all living beings, governs nutrition, growth, and reproduction. The sensitive soul, found in animals, includes these functions while also enabling sensory perception and instinctive responses. The rational soul, exclusive to humans, allows for abstract reasoning, moral judgment, and self-awareness, forming the basis of technical, cultural, and spiritual development. In human beings, these three dimensions—*bios* (biological life), *psyche* (sensitive life), and *nous* (rational-spiritual life)—coexist in a unified whole, enabling them to transcend immediate circumstances and strive for higher goods.

### Faculties of the soul in Aristotelian-Thomistic philosophy

Having considered the essence of the human being through the lens of classical metaphysics, we now turn inward—toward the dynamic structure that animates the human composite: the soul and its faculties^8^. Far from being an abstract notion, the soul in Aristotelian-Thomistic thought is the principle of life and the form of the body, through which man participates in various orders of being. To understand its operations is not to dissect mental processes, but to contemplate the powers by which human life attains knowledge, freedom, and moral depth. These faculties, properly ordered, reveal the unique place of man in the hierarchy of nature: a being capable not only of knowing the world, but of shaping it in accordance with reason and goodness.

In Aristotelian philosophy, the soul’s faculties govern vital and cognitive operations, shaping how living beings engage with their surroundings. Among these faculties, common sense unifies sensory data, allowing individuals to perceive objects as cohesive entities rather than fragmented details. Aristotle considered this function essential for attention, memory, and distinguishing relevant stimuli.

The rational soul, unique to humans, enables higher cognitive functions such as consciousness—the ability to reflect on oneself and assign meaning to experiences—and intentionality, which directs mental states toward external realities. Through memory, for instance, humans create representations of absent objects and transcend the immediate present, forming the basis for personal relationships and the pursuit of purpose.

Additionally, Aristotle distinguished the cogitative faculty in humans from the estimative sense in animals. While animals instinctively assess stimuli for survival, the cogitative faculty allows humans to engage in deliberation, anticipate outcomes, and evaluate ethical implications. This faculty, often termed the “intentional sense of the future,” underpins abstract reasoning and moral agency.

### Intellectual knowledge and the will

While sensory faculties are confined to material conditions, intellectual knowledge is entirely immaterial, enabling humans to grasp the essence of things beyond their physical manifestations. This process begins with abstraction, which extracts universal concepts from particular experiences, allowing the intellect to engage with immutable truths. The intellect operates through conceptualization (forming ideas), judgment (assessing truth), and reasoning (drawing logical conclusions), distinguishing humans from all other beings.

Beyond the intellect, the will is the rational soul’s faculty directed toward the pursuit of the good. Guided by reason yet operating freely, the will is not mere indifference but a capacity for self-determination oriented toward excellence. In Aristotelian-Thomistic ethics, true freedom lies in acting according to human nature, striving for virtue and fulfillment. This integration of intellect and will allows human beings not only to discern the good but also to act upon it, shaping their moral and existential trajectory.

This unique combination of faculties elevates the human being beyond a mere biological existence. As Teilhard de Chardin suggested, humans are not only inhabitants of the cosmos but also its observers, interpreters, and, in many ways, its consciousness (1955). This role entails a responsibility to act in harmony with the natural order, using their higher faculties to contribute to the common good and the advancement of the world in which they are embedded.

It must be emphasized, however, that the human faculties examined throughout this work—such as rationality, language, or moral responsibility—are not prerequisites for dignity, but manifestations of a deeper ontological structure that grounds it. Even when these faculties are impaired or undeveloped, the human being retains their intrinsic worth by virtue of their substantial nature^9^. Dignity, therefore, precedes function, and is revealed, not conferred, through such expressions. This distinction becomes especially important in contexts where human capacities are diminished—whether through age, disability, or illness—yet moral worth must remain unquestioned.

## The meaning of embodiment

Having examined the immaterial faculties that define human interiority, we now descend—without rupture—into the realm of corporeality, not as a separate dimension, but as the very space where the human person becomes visible and operative in the world. In classical philosophy, the body is not a mere vessel or instrument, nor a prison for the soul, but the concrete manifestation of a unified being. It is the soul’s expression in matter, and the matter’s reception of form. To understand the human body is, therefore, to contemplate the unity of spirit and flesh, wherein the person exists not as a ghost in a machine, but as a single substance composed of body and soul. This view offers a corrective to both materialist fragmentation and spiritualist abstraction, grounding dignity not in abstraction, but in the concrete and visible reality of the embodied person. In contrast, contemporary healthcare practices often approach the body in fragmented terms—divided into systems, functions, or pathologies—overlooking its personal and symbolic unity. A philosophical understanding of embodiment restores this unity, reminding health professionals that every bodily reality is already the expression of a singular person.

### The body

The human body, as the material dimension of the person, is intrinsically organized by the soul, which serves as its substantial form [15]. Unlike inanimate objects, which are defined by measurable physical and chemical properties, living bodies—referred to as organisms—possess an intrinsic order directed toward their perfection. Each part is hierarchically structured to contribute to the well-being of the whole, not merely by its location but primarily by its specific function. The complexity of this organization increases with higher forms of life, as seen in the human body, where structures such as the hands and brain are intricately connected, enabling advanced cognitive functions. Similarly, the digestive system is oriented toward nutrition, while the reproductive system facilitates complementarity between the sexes and the continuation of life. This interdependence underscores the organic cooperation among bodily systems, where no part functions in isolation. The nervous system, for instance, not only integrates and coordinates essential processes but also interacts dynamically with the cardiovascular, digestive, and motor systems, ensuring the body’s overall harmony and functionality.

### The body as an expression of the person

Although the human body is closely tied to its biological functions, it also transcends purely material limitations. As Lombo notes, the human body allows individuals to perform activities that are not exclusively physical, such as cognitive, volitional, and spiritual operations. Blanca Castilla emphasizes that the body belongs to the person, serving as an expression of their inner self: “the body is the expression of the person” and, therefore, possesses an indisputable dignity inherent to the person. This integral perspective underscores that the human body is not separate from the person but is a constitutive part of their substantial unity, serving as both the vehicle and the manifestation of their individuality and subjectivity [11].

### The body and human dignity

The complexity and integration of the body’s organic systems and senses reveal a structure oriented toward higher purposes. By transcending purely material functions, the human body expresses the unity and dignity of the person. It is not merely a functional instrument but an essential component of the totality of the human being. This integral view allows us to understand the body not as an object but as part of a substantial composite whose ultimate purpose is the full realization of the person in their physical, cognitive, and spiritual dimensions.

### The sexual dimension of the human person

From the perspective of classical metaphysics, the human person is not a juxtaposition of parts or functions, but a unified substance whose corporeal and spiritual dimensions exist in ordered integration. Within this unity, sexuality is not a marginal or merely biological trait—it is a foundational mode of human being-in-the-world [11]. It reveals the person’s openness to the other and expresses, in bodily form, a deeper metaphysical truth: that the human being is oriented by nature toward relation, gift, and fruitfulness. In contrast to contemporary accounts that either absolutize culture or reduce identity to genetics, the classical tradition reads sexual difference through the lens of finality—a principle which sees in every natural structure a purpose inscribed in its very being. Thus, the sexual dimension cannot be abstracted from the human vocation to communion and love; it is neither accidental nor mutable, but an expression of the person’s essential orientation toward the fulfillment of their nature in relation with another.

Already at the dawn of human existence, sexual difference appears as a fundamental mark of personal identity. Rooted in biological reality and yet irreducible to it, sexuality reveals the human vocation to communion. The biblical expression “male and female He created them”^10^ reflects not merely a biological duality, but an ontological structure essential to the human condition [19]. As the union of body and soul, the human person lives his or her sexuality not only through physiology, but also through reason, will, and interiority.

In the classical tradition, the sexual distinction between man and woman is not accidental or secondary; it is a constitutive aspect of human nature. As in the Aristotelian-Thomistic framework, where form and matter are united in a single substance, so too are soul and body inseparably united in the human person [15]. Thus, the body is not a neutral instrument, but the visible expression of personal identity—including its sexual dimension.

Human sexuality is marked by finality: it is ordered toward love, gift, and fecundity. The distinction between male and female is not merely functional; it discloses a metaphysical openness to the other, and a capacity for relational fulfillment. This view finds resonance in the idea of the person as possessing a spousal structure—a predisposition to self-giving that transcends instinct and points to a higher communion [20].

Even outside the Thomistic tradition, elements of this anthropology find echoes in theological sources. Karl Barth, for instance, highlights the “I–Thou” structure of human existence, seeing in sexual difference a sign of the human vocation to relational life [21]. Though Barth’s framework departs from classical metaphysics—especially in his rejection of the *analogia entis*—his emphasis on the reciprocal nature of human being gestures toward the same existential truth: that the person is not a solitary monad, but one who finds fulfillment in the encounter with the other. Within a metaphysically grounded anthropology, this insight receives its full depth and coherence, preserving the integrity of both nature and vocation.

Contemporary perspectives that seek to detach sexual identity from biological reality often overlook this integral unity. While acknowledging the influence of psychological and cultural elements, the classical view insists that such aspects cannot override the ontological foundation inscribed in the body itself. The richness of human sexuality lies precisely in this unity: it is both biological and spiritual, personal and relational, natural and transcendent [22]. As Julián Marías noted, sexual difference is not merely a biological distinction but a fundamental aspect of the human condition, shaping personal identity and intersubjective [23]. The attempt to suppress sexual difference risks reducing human embodiment to an abstraction. However, this should not be interpreted as a defense of past structures of domination or inequality. Rather, the classical view emphasizes complementarity and mutual recognition, grounded in equal personal dignity and ontological difference. The attempt to suppress the difference between the sexes does not enrich but impoverishes human essence, reducing it to an undifferentiated abstraction.

Properly understood, sexual difference does not create division, but reveals a call to complementarity. It is a gift that enables the formation of deep and enduring bonds, particularly in the context of family and social life. As such, sexuality is not a private construct or fluid expression of the self, but a dimension of the person that participates in the broader order of being—inviting each individual to live in truth, generosity, and communion.

## At the Edge of Nature: human biology and transcendence

The human being, classified biologically within the Animalia kingdom, shares anatomical and physiological structures with other living species. Like all mammals, humans possess systems of reproduction, nutrition, and sensation, marked by complexity and interdependence. Yet, this continuity with the natural world conceals a radical rupture: in the human being, nature becomes conscious of itself.

Despite sharing over 98% of its DNA with other primates [24], the human being transcends biological determinism through the emergence of language, moral conscience, symbolic imagination, and rational reflection. These capacities are not mere developments of animal instinct, but signs of a different order of being - one that is not confined by its environment, but capable of transforming it according to meaning and purpose.

Bipedalism, opposable thumbs, and neural sophistication are not in themselves decisive; what matters is how these serve the human capacity to create tools, express beauty, formulate laws, and seek truth. The human person does not merely react, but chooses; does not only survive, but interprets; does not just reproduce, but loves and educates.

This ontological difference has long been recognized in the Aristotelian-Thomistic tradition, which affirms that the human being is a unity of body and rational soul—a substantial form that organizes matter beyond mere biological function. The body is not a shell or instrument, but the visible expression of an invisible interiority [25].

Human biology, therefore, cannot be understood apart from this inner openness to transcendence. Unlike instinct-driven animals, whose actions are circumscribed by fixed patterns, humans inhabit a vast horizon of possibilities. They may act with generosity or cruelty, seek justice or fall into despair—because their nature is marked by freedom, and their destiny by a vocation to meaning.

In this sense, as Teilhard de Chardin observed, the human being is not merely a product of evolutionary forces, but their self-aware culmination: a being in whom matter has learned to ask about eternity [26].

For the health professions, this insight carries significant ethical and clinical weight. It reminds practitioners that the human person cannot be reduced to genomic data, neural pathways, or evolutionary patterns alone. In every patient encounter, one faces not merely a biological organism, but a subject whose suffering, decisions, and hopes are shaped by a horizon of meaning irreducible to biology.

## The human phenomenon: how humans act

Although humans share nearly all their genetic material with chimpanzees, phenomenological analysis reveals dimensions that distinguish them in a radical way—particularly their intellectual, moral, cultural, and spiritual capacities. These facets not only shape their essence but also unveil the profound complexity of their ontological constitution. Philosophical anthropology, as emphasized by Blanca Castilla, endeavors to grasp this uniqueness precisely through human action (2017). She notes: “While it is true that being is known through its actions, the ontological logic is reversed: if something acts in a particular way, it is because it is endowed with structures that enable that action. Therefore, if a being, under certain conditions, could not act, this would not negate its constitutive being.”

From this perspective, we are invited to contemplate the modalities of human action as outward expressions of an interior reality—a reality that reveals the singular place of the human being within the order of creation.

### Communication and language

One of the most remarkable human abilities is communication through an articulated and complex language. As philosopher Martin Heidegger emphasized, language is not merely a tool for expression, but “the house of Being” [27]. For Heidegger, it is through language that the human being discloses reality and inhabits a meaningful world. This capacity to abstract concepts, transmit knowledge, and establish agreements—such as oaths or promises—reflects not only rational organization but also an openness to others and to transcendence. Language connects humans to both the past and the future, enabling them to construct narratives, perpetuate cultures, and pursue meaning.

Introspection is another essential aspect of the human condition. The ability to reflect on one’s own existence, evaluate the past, and envision the future reveals humanity’s historical dimension. Humans are not merely beings in time but beings who live temporality as a structural dimension of existence. As Heidegger describes in *Being and Time* (2008), human *Dasein* is always already “thrown” into a historical context, and its being is shaped by its relation to time as care and anticipation. The human person does not simply exist in time but projects themselves through it, bearing responsibility for their life as a unified narrative.

In healthcare contexts, this linguistic and temporal depth of the human being has direct implications. To speak with a patient is not merely to inform, but to enter a shared space of meaning, where words bear weight, and silence may speak louder than data. Narrative, testimony, and dialogue are not peripheral to medical care—they are central to the recognition of personhood and to the ethical formation of clinical judgment. A medicine that listens is already a medicine that heals.

### Homo Faber: the Toolmaker

Another defining feature of the human phenomenon is inventiveness. The concept of *homo faber* (man the maker) highlights humanity’s ability to create tools and shape the world [28]. Aristotle referred to the hand as the “instrument of instruments,” emphasizing its role in manipulating objects and crafting artifacts that go beyond immediate needs (2017). This inventiveness, however, is not limited to practical functionality. Humans use their creative abilities to produce artistic, technical, and symbolic works that express inner states, revealing a spiritual dimension unparalleled in other species.

In the field of healthcare, this dimension of *homo faber* manifests both in the design and application of medical technologies, and in the patient’s own creative responses to illness and limitation. The use of tools in medicine should never obscure the fact that the human person is not merely an object of intervention, but a subject capable of adaptation, innovation, and meaning-making. Clinical encounters that honor this inventive capacity foster not only recovery, but the reaffirmation of human dignity in the face of vulnerability.

### Rituals and the search for meaning

Humans are also distinguished by their ritual practices, which transcend biological functionality. The act of burying the dead, for instance, symbolizes respect and suggests a belief in the continuation of existence after death [29]. Such rituals express a recognition of transcendence, a hallmark of humanity’s search for meaning.

Other practices, such as creating music, narratives, ethical systems, and religions, reflect the human effort to understand both themselves and the universe. The capacity to praise, give thanks, and make promises points to a horizon of meaning that transcends physical limitations. As Julian Marías observed, denying this transcendent dimension impoverishes human essence, reducing it to mere biological or sociological functions.

In the healthcare context, the presence—or absence—of ritual and symbolic expression often marks the difference between mere intervention and truly human care. Whether in birth, illness, recovery, or death, patients bring with them narratives, gestures, and symbolic needs that shape how they suffer and how they heal. This is particularly evident in fields such as palliative care and mental health, where the ability to acknowledge and accompany the patient’s search for meaning becomes essential. Recognizing these dimensions allows healthcare professionals to meet not only the physical, but also the existential needs of the person. In this way, the search for meaning becomes not peripheral, but central to ethical and compassionate care.

### Temporality and narrative

Humans are temporal beings whose existence unfolds through time as an essential structure of their being. As Heidegger [30] argued in *Being and Time*, temporality (*Zeitlichkeit*) is not merely a succession of events, but the very horizon within which human existence (*Dasein*) understands itself. The human being does not simply exist “in” time but is constituted by it—projecting itself into the future, returning to its past, and interpreting the present in light of both.

Building upon Heidegger’s insights, Paul Ricœur later developed the concept of narrative identity, showing how individuals make sense of their temporal existence by integrating their actions, experiences, and choices into a coherent story [31]. This narrative construction, though not present in Heidegger’s original existential analysis, complements it by revealing how humans interpret their lives as unfolding journeys shaped by meaning and responsibility.

In applied anthropology, especially within the field of medical humanities, this temporal and narrative dimension becomes essential. Narrative medicine recognizes that listening to the patient’s story is not merely a clinical tool, but a way of acknowledging their personal history, suffering, and hope. Practices such as confession—whether in religious, psychotherapeutic, or personal contexts—serve as vehicles through which individuals confront their deepest truths and reconfigure the meaning of their lives.

In clinical practice, especially in contexts involving chronic illness, mental health, and end-of-life care, acknowledging the temporal and narrative dimension of the patient becomes indispensable. Every diagnosis intersects with a story already in progress; every treatment unfolds within a personal timeline marked by meaning, loss, and expectation. This narrative is not merely psychological or biographical—it is ontological. To be human is to exist temporally and interpretively, woven into a history that reveals identity and calls for responsibility. Health professionals who recognize the patient as a narrative being do more than treat conditions—they accompany lives. This form of care not only respects human dignity, but actively contributes to its restoration in moments of vulnerability.

### Freedom, guilt, and moral responsibility

A fundamental characteristic of human existence is the freedom to choose. Unlike a stone, which “simply exists,” the human being is called to decide—to respond to possibilities and shape their own existence through action and reflection. Freedom, in this context, is not an external privilege, but the very structure of human being.

Yet this freedom is accompanied by what Heidegger calls existential guilt (*Schuld*)—not in the sense of moral transgression, but as an ontological condition. It arises from the realization that one is always already “thrown” into existence, bound to possibilities not chosen, and nevertheless responsible for appropriating them. To be human is to be guilty in the sense of owing oneself an authentic response to being. This guilt is not a feeling, but a structural call to responsibility.

Thus, the human being is not merely one who acts, but one who takes responsibility for their being, confronting the silent demand to become what they are called to be. In this interior space, one engages in an implicit dialogue with what many call God, orienting choices toward truth and meaning that transcend immediate utility.

In medical ethics, the freedom and moral responsibility of the person are not abstract ideals, but concrete realities that shape every clinical decision. Respect for autonomy, informed consent, and shared decision-making all presuppose this ontological structure of freedom. At the same time, patients often face profound experiences of guilt, regret, or responsibility—especially when facing moral dilemmas related to long-term illness, psychological suffering, or the approach of death. Recognizing that these are not mere emotions, but expressions of the person’s moral depth, allows healthcare professionals to offer not only treatment, but accompaniment in the ethical drama of being. Such care, grounded in the dignity of the free and responsible subject, is itself a moral act.

## The gnoseological and axiological dimension of the human being

### Knowledge and value orientation

From a gnoseological perspective, the human being is distinguished by the ability to know not only the sensible world but also transcendental and abstract realities. This openness to knowledge goes beyond the mere apprehension of phenomena, allowing one to grasp the essence of things. Aristotle describes the human intellect as the power to comprehend the universal through the particular, while Thomas Aquinas emphasizes that truth is the ultimate goal of human intelligence. This pursuit of truth, however, does not occur in isolation; it is deeply rooted in an axiological dimension where knowledge is ordered by values. Humans do not merely recognize what *is* but also evaluate what *ought to be*, aligning their conduct with ideals of goodness, justice, and beauty.

Max Scheler highlights that humans are unique in their ability to objectively apprehend values, integrating them into their existence and imbuing them with meaning (2008). Phenomenology, in uncovering the human being’s experiential richness, finds in the gnoseological and axiological horizons an indispensable complement to understanding human uniqueness as a being that seeks truth and orients itself by values that transcend the empirical world.

In healthcare, this orientation toward truth and value is not theoretical—it guides every ethical judgment and therapeutic decision. Clinical discernment involves more than technical accuracy; it requires value-perception: what is good for this patient, in this moment, according to their human dignity. Recognizing that the human person is naturally ordered to truth and goodness reminds professionals that care is not neutral—it is always an ethical act rooted in a hierarchy of values.

### Self-awareness and identity

The human capacity for self-awareness and personal identity is a defining trait that distinguishes the person from all other beings. In *Exodus*, God’s revelation to Moses—“I am who I am”—points to the essence of being as self-subsistent and complete^11^. Though created and contingent, the human person is the only visible being capable of declaring “I am,” a unique ability that Paul VI highlighted as central to human dignity [32]. This self-recognition goes beyond mere biological processes; it is the expression of a reflective capacity that allows individuals to turn inward, perceive themselves as the authors of their actions, and contemplate their own existence [33].

Modern science often attributes consciousness to material brain processes, yet both philosophy and neuroscience acknowledge the difficulty of explaining how physical mechanisms could generate subjectivity and self-awareness [34]. Aristotelian-Thomistic philosophy addresses this challenge by viewing the rational soul as the vital principle that unifies body and spirit into a substantial unity. For Aristotle and Aquinas, consciousness—understood as the capacity to “be a self”—cannot be reduced to materiality but arises from the inseparable union of body and soul, grounding the intrinsic dignity of the person.

However, personal identity is not formed in isolation; it is shaped through relationships and the roles individuals fulfill in society. A person is recognized not only by their self-awareness but also by their interactions with others, assuming roles such as parent, teacher, physician, or friend. These roles are not mere social constructs but expressions of one’s unique identity and ethical commitments. As Shakespeare suggested, life presents individuals as actors on a stage, yet these roles are not superficial masks—they reveal deeper dimensions of personality and vocation. Through these relationships and responsibilities, the human person not only affirms their identity but also actualizes their potential, engaging in a continuous process of self-discovery and self-giving.

The healthcare encounter often confronts identity at its most fragile: in illness, disability, or cognitive decline. Yet even in these states, the person remains a self—capable of presence, relation, and interiority. Recognizing this demands a care that does not reduce the patient to clinical categories, but respects their story, their roles, and their enduring dignity. In this way, health professionals not only treat conditions, but safeguard the personal continuity that illness often threatens.

## Toward the concept of person

The path followed so far has unveiled essential aspects of the human being: the corporeal and spiritual unity, the phenomenology of action, and the gnoseological and axiological dimensions that define human life. These explorations, however, point beyond themselves toward a deeper synthesis-one that transcends faculties and functions to reach the very foundation of human dignity.

Now, we turn to the concept of person, the highest category in philosophical anthropology. Rooted in metaphysical, historical, and theological insights, this notion seeks to express the unrepeatable singularity of each human being as a subject of being, relation, and vocation.

### The concept of person: classical definition and Christian transformation

The classical definition of “person,” inherited from Boethius as “an individual substance of a rational nature,” became foundational in Western thought. It reflected continuity with Augustine’s insights while marking a departure from Platonism by emphasizing individual singularity over universal essence. As Augustine stated in *De Trinitate*, “the term person does not designate the species, but something singular and individual” [35]. This definition was pivotal in theological debates—especially in response to Nestorian and Monophysite positions—by preserving the unity of Christ’s personhood [15].

Thomas Aquinas later refined this notion, asserting that personal dignity stems not from capacities or actions but from a being’s very essence. For Aquinas, “the term person designates what is most noble in the universe, that is, what subsists in a rational nature” (*Summa Theologica*, I, q. 29, a. 3). He synthesized this idea in the expression *subsistens rationale*, emphasizing autonomy, intelligence, and will as defining characteristics of personhood [5]. While Boethius’s definition remained influential, Leonardo Polo^12^ argued that it does not fully capture the existential depth of personhood. For Polo, the person is not merely a rational subject, but a being marked by openness to transcendence and communion^13^ [36].

Historically, the concept of “person” underwent significant semantic and philosophical evolution. In ancient Greece, *anthropos* referred generically to humanity, while Aristotle defined the human as a rational and political animal. The Latin *persona*, derived from the Greek *prosopon*, originally denoted theatrical masks and later gained juridical meaning—identifying individuals with specific civic and legal status [15]. In classical antiquity, individuality was often subordinated to ideals of virtue and citizenship, as seen in Plato’s emphasis on universal Ideas over particular beings.

Christianity introduced a paradigm shift by affirming the intrinsic dignity of the human person as a creature and child of God. This reorientation had practical consequences, such as the emergence of human equality and the emancipation of slaves during the Constantinian and Justinian periods. Christ Himself, rather than the virtuous citizen, became the model of full personhood.

The Church Fathers, especially Gregory of Nazianzus and Gregory of Nyssa, were instrumental in redefining *prosopon* beyond its theatrical connotation. They equated it with *hypostasis*, signifying a subsistent individuality that is distinct yet united to a shared essence. This development enriched the philosophical understanding of personhood by affirming not only individuality but also relationality—the capacity for communion with God and others [37, 38].

The recognition of personhood as an intrinsic and inalienable attribute of the human being was not always evident across history. In various periods, individuals were reduced to social, economic, or political functions, thereby obscuring their transcendent dignity. Theological debates—especially those concerning the Trinity and the Incarnation—proved decisive in deepening and refining the notion of personhood. These controversies led to the development of the concept of *hypostasis* as a subsistent individuality entity [39], unique yet always in relation (Augustine [35]), laying the groundwork for later philosophical anthropology and even the juridical foundations of human rights.

The Christian understanding of the person as a being created *imago Dei* further affirms this intrinsic dignity—not merely as rational or autonomous, but as called to relationality and self-gift. This vocation to communion reflects the inner life of the Divine Trinity, in whose image each human is formed. As Bonaventure elaborated, the person is not merely a “something” among many beings, but a *someone*—a concrete and irreplaceable subject, whose dignity is irreducible. His metaphysical distinctions between *essence* (humanity in general), *substance* (the individual in being), *hypostasis* (the particular identity), and *person* (the unique, self-possessing agent) underscore the depth and mystery of human existence in relation to both others and the transcendent [40].

In this light, theological reflection on personhood is far from abstract speculation: it provides a robust anthropological and ethical foundation for coexistence in a world composed not of interchangeable units, but of irreplaceable persons—each bearing a name, a vocation, and a unique place in the order of being.

### The essential qualities of the person

The act of being, as Lombo and Russo emphasize, is the metaphysical foundation that makes a person truly real. As a subsistent substance, the human person possesses a unique and inalienable act of being that remains constant throughout all stages of development (2020). This subsistence grants essential qualities such as *inalienability*—the person exists in and of itself, irreducible to any external possession—and *irrepeatability*, meaning no individual can be replicated without losing their intrinsic uniqueness. While the human species propagates, the person remains singular, countering pantheistic views that dissolve individuality into nature or divinity. Italian theologian Romano Guardini (1885–1968) encapsulates this by stating: *“No one can inhabit me; I am only with myself”* [41].

The dignity of the person is not merely based on intellectual or volitional faculties but on their irreducible existence. As Kant affirms, the person must always be treated as an end in itself, never as a mere means (2017). This principle rejects both materialistic evolutionism, which reduces humans to instruments of the species, and collectivist ideologies that deny personal autonomy. Though complete in essence, the person is not ontologically self-sufficient in isolation; rather, their fullness is realized in relation to others and the transcendent [42]. This essence is not a closed or static structure, but one that includes the dynamic potential for self-realization within the bounds of its nature.

Beyond completeness, intentionality and relationality further define personhood. Unlike animals, whose behavior is instinct-driven, humans shape their own destiny through deliberate action. This autonomy, however, is not isolation but openness—to reality, to others, and ultimately to God. As Lombo argues, in the person, nature is subordinated to individuality, and actions are truly their own, reflecting self-determination. In this sense, human freedom is not merely an arbitrary exercise but the capacity to choose and act in harmony with the natural order and transcendental values.

In harmony with this vision, Xavier Zubiri emphasizes that the human being possesses what he calls an “open essence”: not because it lacks definition, but because it is oriented toward progressive realization through intelligence, action, and communion [43]. Unlike the rigid and deterministic structures of the universe, human nature is creative, dynamic, and oriented toward growth, progress, and relationships, transcending the biological domain to reveal its uniquely spiritual dimension.

This integral view, which places the person at the center of the cosmos, resonates with both philosophical and theological traditions, from St. Bonaventure to Heidegger [15]. The transition from mere ontic existence to full ontological realization is uniquely human, made possible by the capacity for reflection, self-giving, and transcendence. Thus, relationality is not an incidental property but a constitutive element of personhood, integrating each individual into a broader horizon of meaning and authenticity.

### The ontological and spiritual dimension of the person

In the 20th century, German-Jewish philosopher canonized by the Catholic Church, Edith Stein, offered a masterful synthesis of modern phenomenology and scholastic tradition, illuminating the human being as an indivisible unity of body and spirit. In her work *Finite and Eternal Being*, Stein not only explores the dignity and uniqueness of the human person but also situates the person within the broader ontological horizon of Being^14^ [44]. For her, the person is not merely a subject of experiences but a genuine “universe” of singular meaning, whose identity is fully revealed in its relation to what she calls the Divine Being—understood here as the ultimate ground of meaning and existence. This ontological relation confers an inalienable dignity upon the person, as the acts of knowing and loving—central characteristics of human existence—reflect an intelligible and ordered foundation of reality, often described in metaphysical traditions as the source of all being.

Thus, the person not only possesses a self-aware “I” but also participates in the very structure of Being, achieving its highest realization in openness to the transcendent. Through this integrated vision, Stein enriches the legacy of Christian theology and philosophy while engaging deeply with the existential questions of her time. Her reflections demonstrate that human dignity is intrinsically tied to the call to communion with what she identifies as the Eternal—a call that elevates, rather than alienates, the human being toward a fuller understanding of itself.

If, as Stein shows, human dignity finds its fullness in this relation to the Eternal Ground of Being, such a relationship also unveils a profound paradox: while participating in what transcends us, we remain acutely aware of our own finitude and contingency. German-Jewish philosopher Hans Jonas, in asserting that “being is animated by a must-be that perpetually struggles against non-being,” vividly illustrates this ontological anguish [45]. The human being, though endowed with the act of being, realizes it did not create itself and that its existence is not necessary. Unlike the immutable and necessary laws of mathematics or logic, human existence is marked by contingency: we might not have existed. This awareness, which confronts the creature with an ontological void, propels it to seek the foundation that sustains its existence.

Stein deepens this reflection by showing that the human person not only possesses a “self” but subsists as a unique universe of meaning, whose identity finds its fulfillment in receptivity to the transcendent. The anguish of realizing that we did not create ourselves inevitably points toward a reality that grounds and animates our existence. Thus, the human person, as a hypostasis, experiences individuality as openness to this transcending reality—a call to respond to the mystery of being and the movements of what many traditions refer to as grace.

This interplay between fragility and transcendence transforms anguish into a path of elevation, where the limits of the creature are met by the plenitude of being itself. In this way, human contingency is not merely a sign of limitation but also a bridge leading to communion with the ground of Being—that which sustains, orders, and fulfills our existence.

### Singularity and Personhood: responding to the call of being

The ontological anguish described by Hans Jonas reveals not only the human being’s finitude and contingency but also the awareness of its irreducible singularity. This awareness, marked by the fragility of not having created ourselves and by openness to the transcendent, naturally leads to the question: “Who am I?” This inquiry, which surpasses the essentialist “What am I?” seeks not merely to define the universal nature of the human being but to penetrate the mystery of personal identity and individuality. In this context, a proper name emerges as a powerful symbol of this irreducible singularity. It is not merely a functional identifier but a testament to the fact that each person is unique, called to exist and to occupy an irreplaceable place in the horizon of Being. A name signifies that the person is not an abstraction but a concrete “who,” endowed with a dignity that distinguishes it from all other forms of existence.

Zubiri deepens this distinction by introducing the concepts of personhood (*personalidad*) and personality (*personalidad activa*) [46]. Personhood, which is constitutive and ontological, grants the human being its unique and proper character, whereas personality refers to the dynamic and concrete way in which that personhood is manifested and evolves over time. The anguish of being contingent yet conscious finds its resolution in personhood: it is what empowers the human being to transcend finitude—not by negating it but by embracing it as a path toward a deeper realization of self within the broader structure of meaning and being.

Viktor Frankl complements this vision by asserting that every human being possesses a unique and irreplaceable role, shaped by an unrepeatable combination of predispositions and experiences (2017). This singularity highlights the human person as a being whose existence is rich in meaning, contrasting sharply with categories of beings such as animals or objects, whose individuality is minimal. As Blanca Castilla observes, the acts that shape personality also influence personhood, broadening the ontological understanding of what it means to be a person (2017).

Thus, the initial anguish—born from our finitude and our openness to meaning—finds its fulfillment in personhood. This capacity for self-transcendence allows each person to actualize their uniqueness within a horizon that exceeds mere utility or biological function—a horizon grounded in the search for truth, coherence, and existential depth.

### Personhood and the human encounter in healthcare

The philosophical understanding of the person as a singular, irreducible, and relational being is not merely of theoretical interest. It has concrete and vital implications for the healthcare professions. At the heart of every medical act lies an encounter between persons—each bearing a unique history, a moral interiority, and a vocation that cannot be substituted or reduced to functions or pathologies.

The clinician, too, is not merely a technician or observer, but a person involved in a moral relationship marked by responsibility, compassion, and the search for truth. To recognize the patient as a person means to care not only for a body, but for a being whose identity transcends the clinical chart: someone who suffers, hopes, and seeks meaning even amid illness.

This vision of personhood resists the growing tendency to depersonalize medicine—whether through bureaucratic protocols or impersonal technologies. While innovations such as artificial intelligence may assist in diagnosis or data analysis, they cannot replace the human relationship grounded in mutual presence and ethical responsibility. The person cannot be “processed” like information; they must be received, listened to, and responded to as a mystery.

Understanding the ontological foundation of the person also safeguards the dignity of health professionals themselves. In a world increasingly pressured by efficiency, performance, and depersonalization, the inner life of the caregiver—his or her vocation, conscience, and moral freedom—must be protected and nourished. Only a person can truly care for another person. Only from the depth of personhood can healing, in the full sense, emerge.

## The dignity of the person: between collectivism and relation

The phrase often attributed to Stalin, “The death of one person is a tragedy; the death of millions is a statistic,” though its exact origin remains uncertain, symbolically encapsulates the dehumanizing mindset characteristic of totalitarian regimes. This perspective reduces individuals to mere numbers and statistics, erasing their uniqueness and denying their irreducible dignity. In contrast, the Christian and personalist view of the human person recognizes this uniqueness as inherent and irreplaceable, grounding human dignity in the intrinsic relationship with others and with God. As a counterpoint to totalitarian logic, English poet John Donne (1572–1631), in his *Meditations*, famously stated, “No man is an island,” emphasizing the interconnectedness of all human beings and the value of each singular life. This tension between the depersonalization of totalitarianism and the relational, personalist view underscores the need to reaffirm an ethical framework that upholds the inalienable dignity of the human person, even in contexts that seek to instrumentalize or reduce individuals to numerical abstractions.

In this sense, following Donne’s insight, it becomes undeniable that no human being can be understood in isolation, as interdependence constitutes an essential aspect of human existence. Human life is characterized by a continuous interplay between dependence and autonomy, from conception to death. A person does not create themselves; rather, they are ontologically dependent first on their parents, who provide the material substrate of life through their reproductive cells. Furthermore, there is an ontological relationship with God, who imparts the form or essence of personal existence. As Lombo and Russo observe, the primordial relationships of filiation, maternity, and paternity are constitutive and impose ethical responsibilities (2020). These fundamental relations demonstrate that human existence is essentially relational, always open to others. The presence of the other is, therefore, not an incidental or merely social circumstance but an intrinsic necessity.

It is essential, then, to reconceive the person as a subsistent relation, a *being-with*—a being in coexistence. In this light, Boethius’s classical definition of the person as an “individual substance of a rational nature,” though foundational, does not encompass essential aspects such as freedom and love, which constitute deeper dimensions of personhood [47, 48]. The notion of an isolated person, conceived as an entirely autonomous and self-sufficient entity, is untenable. While the human being is irreducible in their essence, they cannot be understood as a monad closed in upon itself. The term “individual,” though appropriate in logical or juridical contexts, is insufficient to express the fullness of human personality, which includes rationality, relationality, and openness to the transcendent.

As French philosopher Jacques Maritain (1882–1973) notes, modern thought often conflates two concepts that ancient wisdom already distinguished: individuality, which is shared with other forms of life, and personality, which is unique to human beings and implies a singularity open to relationships (2024). Even in seemingly technical processes such as in vitro fertilization, the relational dimension of the person endures—whether biological, ontological, or spiritual. By definition, the person is relational and rational, and their ontological constitution cannot be understood without this dimension of openness to others and to ultimate reality. Thus, relationality is not an accidental attribute but a constitutive and fundamental element of the human being.

Acknowledging the relational nature of human beings allows for a deeper understanding of the person and their intimacy—that “unfathomable and mysterious depth” that Spanish philosopher Jacinto Choza (1944-) calls *ontological depth* [49]. There is always more to be discovered about the person, whose possibilities transcend visible social roles and the limitations imposed by their contingent nature. This ontological mystery invites reflection on human will and freedom, which constitute the dynamic core of personality.

As German philosopher Hannah Arendt (1906–1975) noted, the person, unique and unrepeatable, is a radical enclave of intelligence and freedom (2018). This combination of rationality, singularity, and freedom underscores that the person can never be reduced to a function or a means but must be recognized as an end in themselves—a bearer of a dignity that transcends any utilitarian perspective and places them in direct relation to the transcendent.

This understanding of the person as a relational and irreducible being carries significant implications for healthcare. In environments where individuals are often reduced to cases, diagnoses, or data points, the affirmation of personal dignity resists the fragmentation of care. It reminds us that each patient is not merely an isolated organism or clinical condition, but a unique person embedded in relationships—familial, spiritual, cultural—whose suffering cannot be fully understood apart from these dimensions. Respecting the singularity and relational nature of each person restores the ethical center of medical practice and guards against forms of collectivist logic that would subordinate individuals to institutional or technological imperatives.

## Freedom, responsibility, and the ethical formation of the person

In the 20th century, philosophical reflection on the human person was deeply shaped by existentialism, which emphasized the importance of self-realization as a central dimension of human existence. Beyond material, psychic, and relational aspects, the human being reveals its essence in the capacity to grow, decide, and construct meaning. As Gregory of Nyssa observed, “All beings subject to becoming never remain identical to themselves … to be subject to change is to be born continuously” [50]. This affirmation of dynamism complements rather than negates the ontological foundation of the person. Human nature, while possessing a stable essence, unfolds historically through a process of self-formation that unites freedom and responsibility.

Augustine deepens this notion by asserting that while biological time is outside our control, spiritual maturity depends on free choice [51]. Self-determination, although shaped by biological, psychological, and sociocultural factors, cannot be reduced to them. As Lombo notes, human beings face moral and existential questions—such as “What must I do?”—that transcend mere instinct or adaptation. The worldview one holds profoundly impacts these choices: those who see life as a random process tend to act differently from those who perceive it as oriented toward a transcendent good.

This freedom is not without burden. As Viktor Frankl points out, the human person is not only free but responsible—freedom entails accountability. Our actions express a personal authorship; they arise from a decisive center, a rational and spiritual core that shapes one’s life according to values and purposes [52]. In this light, autonomy is not an end in itself but a vocation to respond to values greater than oneself.

This ethical capacity unfolds within a dynamic interplay between desire, sentiment, and rational duty. As Emmanuel Mounier [48] emphasizes, freedom is never a neutral force—it always implies a moral orientation and personal responsibility. Kant’s categorical imperative gives rational structure to this orientation, insisting that one must act according to maxims that could be willed as universal laws. At the same time, David Hume [53] reminds us that morality also arises from sentiment, revealing that emotional experience is not opposed to reason but part of the moral fabric of human life. Rather than mutually exclusive, reason and sentiment are interwoven dimensions of moral discernment, guiding the person in the search for the good and in the exercise of ethically grounded freedom.

Paul Ricœur [31] contributes to this vision by linking freedom with narrative responsibility. For Ricœur, the self is constituted in time, through a coherent story of choices and relationships. True identity involves assuming responsibility not only before oneself, but also in response to the presence of the other. This contrasts with Sartre’s existentialism, which, denying transcendence, sees freedom as absolute yet anguished—“condemned to be free” [54]. Ricœur instead roots freedom in ethical relation, echoing the biblical insight of Paul the Apostle: “All things are lawful, but not all things are beneficial^15^.”

In classical philosophy, this dynamic is oriented by transcendentals—truth, goodness, and beauty—which constitute the summit of human fulfillment. As Max Scheler [55] argues, values possess ontological objectivity and precede individual emotion. Even in a pluralistic and skeptical age, the ethical calling remains: the challenge is not to deny freedom, but to root it in a hierarchy of meaning that elevates and integrates the human being.

Thus, freedom is not an abstract ideal, nor an arbitrary exercise of will. It is the power to act in accordance with what is true, just, and beautiful. The person matures ethically by aligning their autonomy with values that fulfill their nature. Through this path, the human being transcends limitations and responds to their vocation—freedom not as detachment, but as fidelity to the good.

In healthcare, freedom and responsibility are not only philosophical categories but lived realities. Each clinical decision—whether made by professionals or patients—involves an ethical horizon shaped by values, beliefs, and responsibilities. The professional must exercise discernment not only through protocols, but through moral judgment; the patient, in turn, is often called to make difficult decisions in situations of vulnerability. Respecting the person’s freedom in these contexts does not mean leaving them alone, but walking alongside them—offering clarity, support, and care as they respond to the moral demands of illness, suffering, and healing.

## The ontological Core and the self-transcendence of the person

Although freedom is an essential characteristic of the human person, the ontological core that defines them cannot be overlooked. As Lombo and Russo observe, each individual possesses a permanent ontological identity that sustains their dignity as a personal being. In classical philosophy, substance—understood as the unity of matter and form, body and soul—is not an inert substrate but the source of autonomy and the act of being. The soul, conceived as the substantial form of the human being, is naturally oriented toward its perfection, an inclination derived from the individual’s essence. However, in humans, this intrinsic propensity is mediated by intelligence, values, and freedom, allowing for a flexibility of choices and actions that distinguishes them from the rigid instincts of animals. As the authors emphasizes, the will is a spiritual inclination directed toward the good recognized by the intellect, harmoniously integrated into human essence. Thus, freedom is neither an abstraction disconnected from reality nor unlimited: it is conditioned by circumstances, values, and the pursuit of a rationally appropriate good, as Aquinas pointed out. This capacity to deliberate, transcend oneself, and orient existence toward higher ideals is what characterizes the human person and grounds their dignity, as elucidated by 20th-century personalist thinkers.

In this sense, as Russian philosopher Nicolai Berdyaev (1874–1948) highlights, the human person transcends merely biological or psychological categories, belonging instead to an ethical and spiritual order [56]. This conception underscores that human dignity is not reducible to the sum of observable characteristics but finds its true essence in the metaphysical depth of being. Even when an individual does not fully achieve self-transcendence, their dignity remains firmly anchored in an ontological substrate that grounds their identity and intrinsic worth. It is this metaphysical root that ensures the person’s irreducibility and centrality within the moral order.

Although conditioned by biological, psychological, and sociocultural factors, human freedom is the means through which the person seeks the realization of the good and the discovery of truth. This freedom should not be conceived as an arbitrary exercise of will but as a path that ultimately transcends individual limitations and connects the person to a broader horizon of meaning and value. Thus, the pursuit of the good and the truth is not merely an ethical aspiration but an expression of the spiritual nature of the person. In their capacity for self-transcendence, the person projects themselves beyond their own limits, engaging with higher realities. In this way, self-transcendence is not an unattainable ideal but a potential manifestation of human freedom, oriented by principles that elevate the person above the contingent and ephemeral, reaffirming their dignity and transcendent purpose.

In clinical contexts, particularly in end-of-life care or in patients with severe cognitive impairment, the affirmation of an ontological core of personhood resists the temptation to reduce individuals to their current functional capacities. Even when communication, autonomy, or awareness are compromised, the person remains a bearer of dignity. Recognizing this foundational identity guides healthcare professionals to preserve respect, compassion, and ethical integrity, especially when curative interventions are no longer possible. In such moments, care becomes an act of fidelity to the irreducible worth of the person—beyond utility, performance, or prognosis.

## Transcendental anthropology and the vocation to the infinite

Building upon the ontological foundation of the human being, we now turn to the transcendental dimension of personhood—not as a speculative addendum, but as an intrinsic structure of the human mode of being. The capacity for self-transcendence, evident in moral action, symbolic thought, and the pursuit of truth, suggests that the human person is not exhausted by empirical determination or functional activity. Rather, the human being appears as a *being-in-relation* to that which exceeds them—a horizon of meaning irreducible to the sum of material, psychological, or social elements.

This perspective belongs to what may be termed transcendental anthropology: a philosophical endeavor to account for the human being not merely in terms of substance (body and soul), but also through the lens of their orientation toward *Being itself*—the intelligible totality within which existence finds its coherence. Unlike the transcendental anthropology of Kant, focused on the a priori conditions of knowledge, this approach—developed notably by Leonardo Polo—seeks to unveil the ontological openness proper to human nature. For Polo, the person is not an intramundane object among others, but a reality constituted in its *aperture* to Being, whose act is both singular and free (2019). This openness is not an accidental trait, but a structural dimension of the human being as *coexistent with meaning*.

Philosophers such as Edith Stein have emphasized this constitutive depth. In her synthesis of phenomenology and metaphysics, Stein describes the person as a dynamic unity of body, soul, and spirit—whose being is marked by an intrinsic receptivity to that which transcends the empirical (2002). Rather than reducing the person to functions or states of consciousness, she identifies in human subjectivity an ontological depth that grounds both selfhood and moral responsibility.

This orientation toward the transcendent—understood here in ontological, not theological terms—has also been highlighted by figures such as Bruno Snell, who in *The Discovery of the Mind* reveals the emergence of human self-consciousness as the birth of the spiritual in Western thought [57]. The human being, in this view, is not merely a rational animal, but a *symbolic being* capable of contemplating the eternal, formulating ideals, and seeking meaning beyond necessity.

Viktor Frankl’s analysis of the noetic dimension further reinforces this interpretation (2017). In his existential psychology, the search for meaning emerges as a primary human drive—not reducible to pleasure or survival. Frankl demonstrates that even suffering, when integrated into a coherent framework of meaning, can become a path to dignity. This search presupposes a capacity for self-transcendence that expresses the spiritual structure of the person—an openness to that which cannot be controlled, consumed, or reduced.

In metaphysical terms, Xavier Zubiri articulates this distinction by contrasting the closed essence of the cosmos with the open essence of the person (2008). Unlike natural entities governed by fixed determinisms, the human being is marked by a unique act of being—an act that unfolds through freedom, intelligence, and historical self-realization. This act, while conditioned, is not determined; it bears within itself a *transcendental potentiality* that situates the person at the intersection of immanence and transcendence.

Leonardo Polo expands this vision by distinguishing three levels of being: the being of the cosmos (determined and unified), the being proper to each person (free and singular), and the fullness of Being as such (2019). While the latter has traditionally been identified with the Absolute in metaphysical traditions, this article does not rely on theological categories but rather emphasizes the philosophical significance of this *ontological gradation*. What matters is that the human being, unlike other entities, *participates* in being through a free act that cannot be reduced to biology, psychology, or culture.

This participation implies that the person is not statically defined by essence alone, but dynamically realized through freedom, value-orientation, and self-determination. As Blanca Castilla observes, the human act of being is unrepeatable and personal—it is not merely an instance of a species, but a singular presence, open to truth and responsibility(2017). This ontological uniqueness, far from isolating the person, situates them within a network of relations that reveal the mystery of existence as a shared, yet singular, journey.

In this light, human dignity is not a product of social recognition or cognitive capacity, but a metaphysical given—rooted in the very structure of personal being. The transcendental orientation of the person testifies to a depth that no empirical description can exhaust. Even when diminished in function, the human being remains a subject of meaning, of value, of *being-toward*.

Hence, transcendental anthropology does not appeal to theological revelation, but to a philosophical insight: that to be human is to stand before Being with the capacity to question, to respond, and to commit oneself to what is true, good, and beautiful. This vocation to the infinite, however it is interpreted, belongs not to the margins of human life, but to its very center.

In medical contexts, especially those dominated by efficiency, prognosis, or protocol, this transcendental dimension is often overlooked. Yet the patient is not merely a body to be treated or a mind to be stabilized, but a person whose being is open to meaning—even in silence, in suffering, or in apparent passivity. Recognizing this spiritual and transcendental orientation invites healthcare professionals to approach the patient not only as a recipient of interventions, but as a subject still capable of question, trust, and hope. This is increasingly acknowledged in international guidelines: the World Health Organization and the American Medical Association both emphasize the importance of addressing the spiritual dimension in healthcare, particularly in contexts of chronic illness, palliative care, and mental health^16^. In this light, care becomes more than treatment—it becomes an act of witnessing the irreducible mystery of the person.

## Contrasting ontological and functionalist perspectives: toward a renewed foundation for ethical action

The tension between ontological and functionalist views of the human person is not merely theoretical, but exerts decisive influence in the field of bioethics. Functionalist accounts, as exemplified by Peter Singer and Michael Tooley, reduce personhood to the exercise of certain cognitive capacities—such as self-awareness, rational agency, and the possession of future-oriented desires. Within this framework, infants, persons with advanced dementia, or those in persistent vegetative states are denied full moral status—not for lack of humanity, but for lack of function. Singer [1], for instance, contends that some non-human animals may possess greater moral standing than newborn humans, due to their higher cognitive abilities. Tooley [2] similarly argues that the right to life depends upon the individual’s capacity for self-consciousness and psychological continuity.

This perspective radically fragments the classical unity of the human being. It transforms dignity from an intrinsic metaphysical attribute into a contingent status, arbitrated by neurological thresholds. Such a model risks legitimizing forms of discrimination against the most vulnerable—those whose capacities are diminished or undeveloped—and ultimately reduces ethical worth to measurable output.

Materialist thinkers go even further by denying the existence of any ontological substrate beyond the physical. Daniel Dennett [58] dissolves the notion of the self into a narrative abstraction—a “center of narrative gravity”—with no enduring reality. Patricia Churchland [59] dismisses the soul as a pre-scientific construct, asserting that ethical reflection must be grounded in the empirical findings of neuroscience. Within this paradigm, the human being becomes fully explicable in terms of biological processes, and ethical reasoning is subordinated to the mechanics of brain activity.

In parallel, some bioethicists have questioned whether the notion of “human dignity” adds any meaningful content to ethical deliberation. Ruth Macklin [12] argues that dignity is a “useless concept,” proposing instead a framework based entirely on autonomy, beneficence, and justice. But this procedural triad, in the absence of an ontological foundation, fails to account for the inalienable worth of human life—particularly in situations where autonomy is diminished or absent.

By contrast, the ontological view affirms that dignity precedes all functionality. Rooted in the metaphysical structure of the human person—an individual substance of a rational nature—it recognizes value in every human being regardless of age, capacity, or condition. This vision sustains the moral imperative to care, to protect, and to affirm life even in its most fragile expressions. It is not dependent on fluctuating standards of ability, but grounded in what the human being *is*. This perspective does not depend on religious revelation, but arises from a philosophical understanding of the human being as an irreducible unity of body and spirit—whose worth is not contingent upon external recognition or internal capacity.

This understanding aligns with current international health guidelines that call for holistic care, recognizing not only the biological but also the spiritual and relational dimensions of the patient. In an age increasingly shaped by technological utilitarianism and a narrow focus on performance, this metaphysical anthropology offers a deeper and more humane foundation for ethical action. It affirms that the call to justice is not merely procedural but ontological; not only about fairness, but about recognizing in every human being a reality that transcends utility [60, 61].

This conviction found concrete resonance in the work of Paul Tournier, whose *Medicine of the Person* movement emphasized the need to treat patients not merely as biological organisms, but as persons—embodied, spiritual, relational beings^17^. For Tournier, clinical care devoid of attention to the interior life and existential meaning of the patient was not only insufficient, but ethically impoverished. This vision illustrates how ontological insights into the human person can—and must—shape medical practice that is both technically competent and morally sound. His approach anticipated many current developments in person-centered and narrative medicine, which seek to restore the ethical and existential dimension of care by viewing the patient not as a malfunctioning body, but as a whole person—capable of meaning, relationship, and transcendence.

## Conclusion

This study has sought to recover the ontological depth and transcendent foundation that define the singular reality of the human person. From the Aristotelian-Thomistic vision of man as a substantial unity of soul and body, to the contemporary developments found in the works of Edith Stein, Xavier Zubiri, and Leonardo Polo, one truth becomes manifest: human dignity is not a derivative status conferred by function or condition, but a metaphysical given rooted in the very structure of being.

The person is not a closed system, reducible to biological data or psychological mechanisms, but a being constituted in relation—relation to self, to others, and to the Absolute, understood here as the ultimate ground of meaning and being. As such, the human person bears an interior openness to the infinite, a radical freedom, and a call to self-transcendence. These are not accidents of development but essential notes of personhood, grounding a dignity that no circumstance can nullify.

In this light, ethics—especially in the realm of healthcare—must proceed from a vision that does not fragment the human being into compartments of function or utility. The body, the affective life, the spiritual hunger for meaning: all must be recognized in their inseparable unity. Attending to the patient is not merely the application of technique, but the reception of a mystery—of someone who carries within themselves the echo of the eternal.

This anthropological foundation is not a theoretical luxury, but a practical necessity in contexts such as end-of-life care, psychiatric practice, chronic illness management, and the ethical dilemmas posed by emerging technologies. A renewed metaphysical understanding of the person equips healthcare professionals to approach the patient not as a malfunctioning system, but as a subject of meaning—capable of narrative, relationship, suffering, and transcendence.

Ultimately, reflecting on the nature of the human person is not a merely speculative endeavor. It constitutes a moral responsibility and a necessary foundation for ethical discernment. In the clinical context, this anthropological depth is not a theoretical luxury but a practical necessity. By recovering a coherent and unified vision of the human being, we can foster a medical practice that is not only technically competent, but also ethically grounded and genuinely humane.

## Data Availability

Not applicable.

## Footnotes

1 Although Engelhardt later embraced Eastern Orthodox Christianity, his bioethical framework consistently upheld a functionalist definition of personhood, grounded in analytical philosophy and libertarian ethics. This article engages that view critically, in favor of a metaphysical and ontological account of human dignity.

2 Genesis 1:27.

3 Language assistance and editorial support for this article were provided using AI tools. However, all ideas, structure, argumentation, and conceptual content are entirely the result of the authors’ original research, reflection, and responsibility.

4 *Metaphysical* refers to that which pertains to metaphysics, the branch of philosophy concerned with the fundamental nature of reality, being, and existence. It often deals with concepts that transcend the physical and empirical, such as the nature of the soul, the relationship between mind and matter, and the existence of ultimate causes or principles beyond sensory perception.

5 In Thomistic metaphysics, the concept of *actus essendi* (“act of being”) refers to the metaphysical act that makes a being actually exist. Distinct from essence (what something is), *actus essendi* is the act by which a being is—a participation in the very act of existence. For Thomas Aquinas, it is this act that confers ontological dignity, grounding the irreducibility and metaphysical depth of the human person. See: Thomas Aquinas, *De Ente et Essentia* [14]; Lombo & Russo [15].

6 Jeremiah 1:5.

7 “Mode of being” refers to the specific way in which a being exists or manifests itself. In philosophical discourse, particularly in existentialism and metaphysics, this concept is used to distinguish between different levels or structures of existence. For example, Martin Heidegger differentiates between the mode of being of inanimate objects, living beings, and human existence (*Dasein*), emphasizing that each has a distinct way of relating to the world. Similarly, Aristotelian and Thomistic traditions recognize different modes of being, such as the substantial being of a person versus the accidental being of qualities or relations.

8 **Aristotelian-Thomistic Philosophy** refers to the philosophical tradition that integrates Aristotle’s metaphysical framework with the theological and philosophical insights of Thomas Aquinas. This synthesis, developed in the Middle Ages, provides a comprehensive understanding of reality, particularly in the areas of metaphysics, epistemology, and ethics. It upholds a hylomorphic view of the human being (as a unity of body and soul) and affirms the existence of final causality, rational intellect, and natural law as foundational principles of human nature and morality.

9 In classical metaphysics, particularly in the Aristotelian-Thomistic tradition, the dignity of the human being is rooted in their *esse*—the act of being—and their substantial nature, not in the actualization of specific operations (*actus humani*). Faculties such as intellect and will are considered *potentiae ordinatae ad esse personae*, that is, ordered to the being of the person, but not conditions for it. Their absence or dormancy does not diminish the ontological status or moral worth of the human individual.

10 Genesis 1:27.

11 Exodus 3:13–14.

12 Leonardo Polo (1926–2013) was a Spanish philosopher who developed a transcendental anthropology rooted in classical metaphysics. His thought emphasizes the act of being as proper to the person and their openness to transcendence.

13 In contemporary bioethics, figures such as Peter Singer and Michael Tooley argue for a distinction between ‘humans’ and ‘persons’ based on rational and moral capacities. However, this paper adopts a more integrated understanding of personhood, aligning it with ontological dignity rather than functionalist criteria.

14 “Being” refers to the fundamental reality or essence of existence, as distinguished from particular beings or entities. In philosophical discourse, especially in metaphysics and existential thought, “Being” denotes the ultimate ground of reality, encompassing both existence itself and the principles that underlie it. Thinkers such as Martin Heidegger and Thomas Aquinas explored Being as the foundational question of philosophy, seeking to understand not just what things are, but what it means to be.

15 1 Corinthians 10:23. “All things are lawful for me, but not all things are expedient”.

16 See: World Health Organization. *Integrating the Spiritual Dimension into Palliative Care: A WHO Guide*. Geneva: WHO, 2021. See also: American Medical Association (AMA), *Spirituality in Clinical Practice*, JAMA Network, 2003–2020.

17 The references to Paul Tournier in this section are conceptual syntheses based on the central theses of *The Healing of Persons* (1965) and *Medicine of the Person* (1983). These are not verbatim quotations but thematic paraphrases reflecting Tournier’s anthropological and clinical philosophy—particularly his emphasis on integrating bodily, spiritual, and relational dimensions in medical care.

## References

[1] Singer, P. In: Practical ethics. 3rd ed. Cambridge University Press; 2011

[2] Tooley M. “Personhood”. In: Kuhse H, Singer P, editors. A Companion to Bioethics. Oxford: Blackwell; 1998. p. 118–126.

[3] Engelhardt HT. The foundations of bioethics. 2nd ed. Oxford: Oxford University Press; 1996.

[4] Boethius S. The consolation of philosophy. London: Penguin Classics; 1999.

[5] Aquinas T, Summa Theologiae. Edited by The Aquinas Institute,. 1st ed. Steubenville: Emmaus Academic; 2012.

[6] Reale G. A History of Ancient Philosophy II: Plato and Aristotle. 1st ed. New York: State University of New York Press; 1990.

[7] Kant I. The metaphysics of morals. 2nd ed. Cambridge: Cambridge University Press; 2017.

[8] General Assembly of the United Nations. Universal Declaration of Human Rights. Paris; 1948.

[9] Lander Es, et al. Initial sequencing and analysis of the human genome. Nature. 2001;15(409):860–921 10.1038/35057062 11237011

[10] Burrow JR. Personalism: A critical introduction. Des Peres: Chalice Pr; 1999.

[11] Cortázar B, Castilla De, González Ginocchio D. The notion of person and a transcendental anthropology, from Boethius to Polo. Spanish in Miscelánea Poliana. 2017;4:81–117.

[12] Macklin R. Dignity is a useless concept. BMJ. 2003;327:1419–1420. 14684633 10.1136/bmj.327.7429.1419 PMC300789

[13] Aristotle. The metaphysics. Amherst: Prometheus; 1991.

[14] Aquinas T. On being and essence. Translated by Maurer A. Toronto: Pontifical Institute of Mediaeval Studies; 1968.

[15] Lombo, JA and F Russo. Antropologia Filosófica: Uma Introdução. 1st ed. Sumaré: Cultor de Livros; 2020

[16] Aristotle, Physics. Indianapolis: Hackett Publishing Co; 2018.

[17] Aristotle. The Nicomachean ethics. Hertfordshire: Wordsworth Editions; 1996.

[18] Aristotle. De Anima (On the Soul). Scotts Valley: CreateSpace Independent Publishing Platform; 2017.

[19] Nyssa GO. On the making of man. Scotts Valley: CreateSpace Independent Publishing Platform; 2013.

[20] Pope John Paul II. The theology of the body: human love in the divine plan. Pauline Books & Media; 1997.

[21] Barth K. “Vol. III The Doctrine of Creation”. In: Bromiley GW, Torrance TF, editors. Church Dogmatics. Edinburgh: T&T Clark; 1961.

[22] Secretan, P. Introdução Ao Pensamento de Xavier Zubiri. São Paulo: É Realizações; 2014.

[23] Marías, J. Persona. Madrid: Alianza Editorial SA; 2007.

[24] Waterson RH, Lander ES, Wilson RK, The chimpanzee sequencing and analysis consortium. Initial sequence of the chimpanzee genome and comparison with the human genome. Nature. 2005;437(7055):69–87. 10.1038/nature04072 16136131 10.1038/nature04072

[25] De Cortázar, Transcendental anthropology and the foundation of human dignity. J Polian Stud. 2014;105–119.

[26] Chardin, T. Le Phenomene Humain. Paris: Editions du Seuil; 1955.

[27] Heidegger M. On the way to language. New York: Harper & Row; 1971.

[28] Arendt H. The human condition. 2nd ed. Chicago: The University of Chicago Press; 2018.

[29] Durkheim E. The elementary forms of religious life. Oxford: Oxford University Press; 2008.

[30] Heidegger, Martin. Being and time. New York: Harper Perennial; 2008.

[31] Ricoeur, Paul. Oneself as another. Reissue. Chicago: University of Chicago Press; 1995.

[32] Pope Paul VI. Gaudium et Spes In: Pastoral Constitution on the Church in the Modern World. Washington: United States Catholic Conference; 1965.

[33] Augustine. Confessions. London: Penguin Books; 1961.

[34] Chalmers DJ. The conscious mind: In search of a fundamental theory. Oxford: Oxford University Press; 1997.

[35] Augustine. The Trinity (Works of Saint Augustine: A Translation for the 21st Century). 2nd ed. New York: New City Press; 2012.

[36] Polo L. Presente Y Futuro Del Hombre. 3rd ed. Madrid: Ediciones Rialp S.A.; 2019.

[37] Gregory of Nazianzus, Wickham L, Williams F. On god and Christ: The five theological orations and two letters to cledonius. In: Behr J, editor. Popular Patristics Series, No. 23. New York: St Vladimir’s Seminary Press; 2011.

[38] Nyssa GO, Scharff P. Four dogmatic Treatises: On the holy spirit, on the holy trinity, and of the godhead of the holy spirit, on not three gods, on the faith. Edited by Doone J. Independently published; 2024.

[39] Basil the Great. On the Holy Spirit: St. Basil the Great. Popular Patristics No. 42. New York: St Vladimir’s Seminary Press; 2011.

[40] Gilson E, Sheed FJ. The philosophy of saint bonaventure. Rhode Island: Cluny Media; 2022.

[41] Guardini, R. Persona E Libertà. Saggi Di Fondazione Della Teoria Pedagogica. Brescia: Morcelliana; 2023

[42] Lombo JA, Giménez Amaya JM. La Unidad de La Persona: Aproximación Interdisciplinar Desde la Filosofía Y La Neurociencia. 1st ed. Navarra: EUNSA, Ediciones Universidad de Navarra; 2013.

[43] Zubiri X. El Hombre y Dios. Madrid: Alianza Editorial SA; 1984.

[44] Stein E. Finite and eternal being: An attempt at an ascent to the meaning of being. Washington, DC: ICS Publications; 2002.

[45] Jonas H, Jonas E, Vogel L. The phenomenon of life: Toward a philosophical biology. 1st ed. Evanston: Northwestern University Press; 2001.

[46] Zubiri X. Sobre la Esencia. 1st ed. Madrid: Alianza Editorial SA; 2008.

[47] Maritain J. Integral humanism: Temporal and spiritual problems of a New Christendom. Rhode Island: Cluny Media; 2024.

[48] Mounier E. Personalism. 1st ed. Notre Dame: University of Notre Dame Press; 1989.

[49] Choza J. Antropología Filosófica. Las Representaciones Del Sí Mismo. Madrid: Biblioteca Nueva; 2002.

[50] Nyssa GO, Malherbe AJ, Ferguson E. Gregory of Nyssa: The Life of Moses. San Francisco: HarperOne; 2006.

[51] Augustine. On free choice of the will. Indianapolis: Hackett Publishing Co; 1993.

[52] Frankl, V. Man´s search for meaning. Boston: Beacon Press; 2017.

[53] Hume D. A treatise of human nature: Being an attempt to introduce the experimental method of reasoning into moral subjects. Springfield: Grapevine; 2024.

[54] Sartre JP. Being and nothingness. New York: Washington Square Press; 1993.

[55] Scheler, M. The human place in the cosmos. Evanston: Northwestern University Press; 2008.

[56] Berdyaev N, Duddington N. The destiny of man. 3rd ed. Semantron Press; 2009.

[57] Snell B. The discovery of the mind. New York: Dover Publications; 2011.

[58] Dennett D. Consciousness Explained. London: Little, Brown and Company; 1991.

[59] Churchland P. Neurophilosophy: Toward a unified science of the mind-brain. Cambridge: MIT Press; 1986.

[60] Tournier P, Gilmore JS. Medicine of the person. London: SCM Press; 1983.

[61] Tournier P, Hudson E. The healing of persons. New York: Harper & Row; 1965.

